# Phylogenetic analysis and virulence determinant of the host-adapted *Staphylococcus aureus* lineage ST188 in China

**DOI:** 10.1038/s41426-018-0048-7

**Published:** 2018-03-29

**Authors:** Yanan Wang, Qingyun Liu, Qian Liu, Qianqian Gao, Huiying Lu, Hongwei Meng, Yihui Xie, Qian Huang, Xiaowei Ma, Hua Wang, Juanxiu Qin, Qiong Li, Tianming Li, Qiang Xia, Min Li

**Affiliations:** 1 Department of Laboratory Medicine, Renji Hospital, School of Medicine, Shanghai Jiaotong University, Shanghai, 200127 China; 2 Key Laboratory of Medical Molecular Virology of Ministries of Education and Health, Institutes of Biomedical Sciences and Institute of Medical Microbiology, School of Basic Medical Sciences, Fudan University, Shanghai, 200032 China; 3 Department of Liver Surgery, Renji Hospital, School of Medicine, Shanghai Jiaotong University, Shanghai, 200127 China; 4 Faculty of Medical Laboratory Science, School of Medicine, Shanghai Jiaotong University, Shanghai, 200025 China

## Abstract

*Staphylococcus aureus* (*S. aureus*) is an important pathogen of humans and livestock species, but an understanding of the clonal distribution of *S. aureus* causing different host-species infections in the same geographical environment and within the same period is lacking. By characterizing infections caused by *S. aureus* in bovine, pediatric, and adult patients in Shanghai, China, between 2012 and 2014, we identified methicillin-sensitive *S. aureus* (MSSA) ST188 as the major lineage causing infections in multiple host species. Whole-genome sequencing and phenotypic analyses demonstrated that ST188 might evolve from livestock, and there was no significant genomic or virulence difference between ST188 isolated from livestock and humans. The virulence of ST188 is related to its adhesion and nasal colonization ability. This result is in accord with the strong epithelial cell adhesion and biofilm formation properties of ST188. Furthermore, the adhesion- and biofilm-formation-related genes are present in multiple copies and exhibit significantly increased expression in ST188. In conclusion, *S. aureus* ST188 is the major lineage causing human and livestock infections in Shanghai, China. Due to its high expression of the factors associated with bacterial adhesion and biofilm formation, ST188 has the ability to colonize and infect different host species.

## Introduction

*S. aureus* is a major human and economically important livestock species pathogen that is responsible for a variety of infectious diseases^1,2^. *S. aureus* can cause skin and soft tissue infections, pneumonia, bacteremia, and endocarditis in human beings^1^, and it is also considered one of the most relevant pathogens causing skin abscesses, mastitis, and skeletal infections in livestock^3^. Current typing methods, such as multi-locus sequence typing (MLST), have been applied to examine the population structure of *S. aureus* strains with respect to their specificity for infecting humans or animals. The majority of human and animal *S. aureus* isolates belong to a small number of host specificity clones^4,5^.

*S. aureus* mainly causes hospital-associated infections in predisposed individuals. In China, as in most Asian countries, the multiple-antibiotic-resistant strains ST239 and ST5 are of predominantly human hospital-associated methicillin-resistant *S. aureus* (HA-MRSA) lineages^6,7^. These two lineages account for nearly 90% of all HA-MRSA isolates in Chinese hospitals^6^. Recent studies have demonstrated that ST239 displays adaptive evolution with attenuated virulence for successful colonization in the environment with high antibiotics pressure. ST239 has also acquired specific molecular determinants to promote nasal colonization, immune evasion and virulence via horizontal gene transfer^8^. Neither ST239 nor ST5, which exhibit host specificity and restriction, have been isolated from livestock animals in China^9^. In contrast, emerging community-associated *S. aureus* (CA-SA) strains have collectively demonstrated increased virulence potential enabling the infection of otherwise healthy people outside of hospital settings^10^. The dominant CA-SA strain in the United States is the highly virulent USA300 (ST8), while the predominant Asian CA-SA lineage is ST59^11^. ST59 exhibits more pronounced virulence than the geographically matched HA-MRSA clones ST5 and ST239^12^. By high expression of core genome-encoded virulence determinants, CA-SA has acquired high-virulence phenotypes^13^.

Although nearly all *S. aureus* lineages are host-specific, there are strains that have a broader host tropism^14^. Livestock-associated *S. aureus* (LA-SA) ST398 was originally reported among pigs and pig farmers in the Netherlands in 2003, and it has been identified as the most pandemic LA-MRSA in Europe and North America^15^. However, in the Americas, ST398 is now also recognized as a causative agent of infections in humans living in animal-free environments, suggesting host-adaptive diversification of ST398^16^. Whole-genome sequencing (WGS) revealed this difference in its genetic composition, which was associated with significantly enhanced adhesion of human ST398 isolates and transmission among humans^17^. Although we have observed a considerably high rate of CA-SA infections caused by highly virulent ST398 in China^18^, the predominant LA-MRSA lineage in China is ST9^15^, and most LA-ST398 infections in China have been reported to be MSSA infections^9^. This incongruent distribution implies that the clonal distribution of *S. aureus* causing different host species infections has considerable geographical variation.

To analyze the clonal distribution of *S. aureus* causing infections in different host species within the same geographical environment and within the same period, we investigated bovine, pediatric, and adult patient infections caused by *S. aureus* in Shanghai, China, between 2012 and 2014. WGS and in vivo infection models were used to clarify the characteristics of host-adaptable *S. aureus*.

## Results

### *S. aureus* ST188 is the major lineage causing infection in humans and livestock in Shanghai, China

**Fig. 1:**
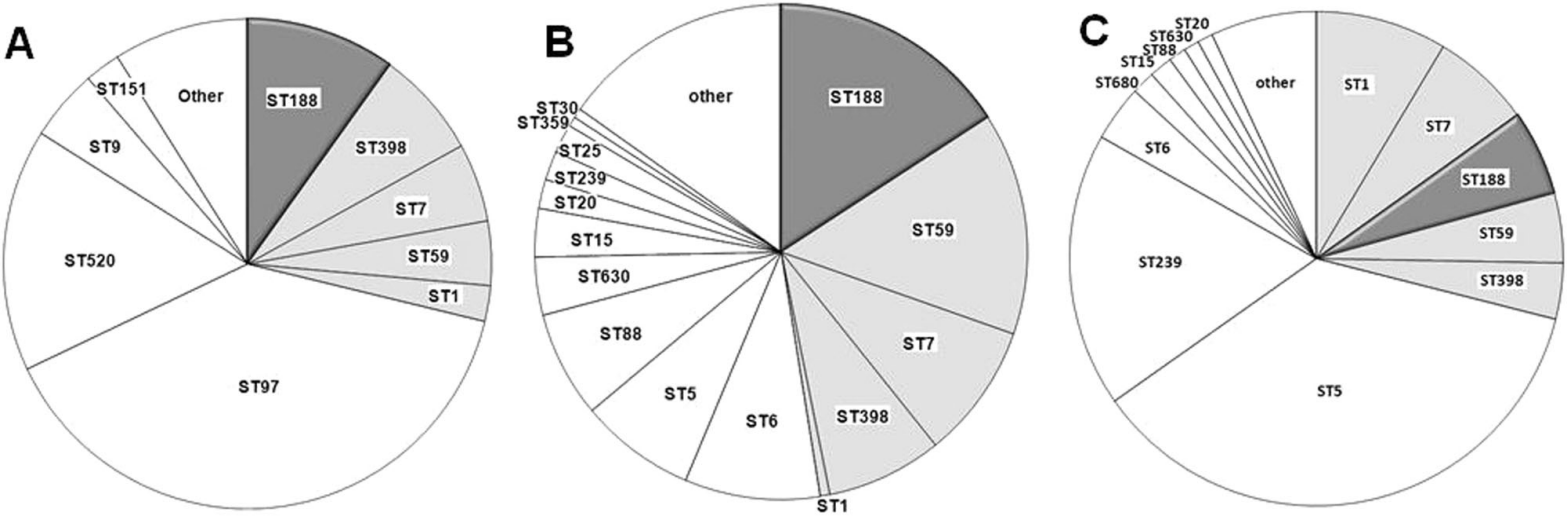
Clonal distribution of *S. aureus* causing infections of different host species in Shanghai, China, between 2012 and 2014. **a** Clonal distribution of *S. aureus* causing bovine mastitis. **b** Clonal distribution of *S. aureus* causing infections in pediatric patients. **c** Clonal distribution of *S. aureus* causing infections in adult patients. Clones marked in gray: sequence types causing infections in both humans and livestock

The clonal distribution of *S. aureus* causing infections in different host species showed that many *S. aureus* lineages were host-specific (Fig. 1). The predominant lineages causing livestock-specific infection were ST97 (39.2%) and ST520 (16.0%) (Fig. 1a). The major lineages causing human-specific infections were ST5 (36.3%) and ST239 (18.0%) (Fig. 1c). However, several lineages showed the capacity to cause disease in multiple host species: ST188, ST398, ST7, ST59, and ST1 (Fig. 1a–c). Among them, ST188 was the major host-adapted lineage, causing 9.9% (21/212) of livestock-associated infections, 15.7% (25/159) of pediatric patient infections and 5.6% (44/791) of adult patient infections; moreover, t189 was of the epidemic *spa* type (>80%) in all types of host-associated ST188 (Supplementary Figure 1A). The *Agr* type was detected in all *S. aureus* ST188, and both human-associated (100%) and livestock-associated (100%) ST188 carried *agr*-group I.

### Phylogenetic analysis of host-adapted ST188 isolates

**Fig. 2:**
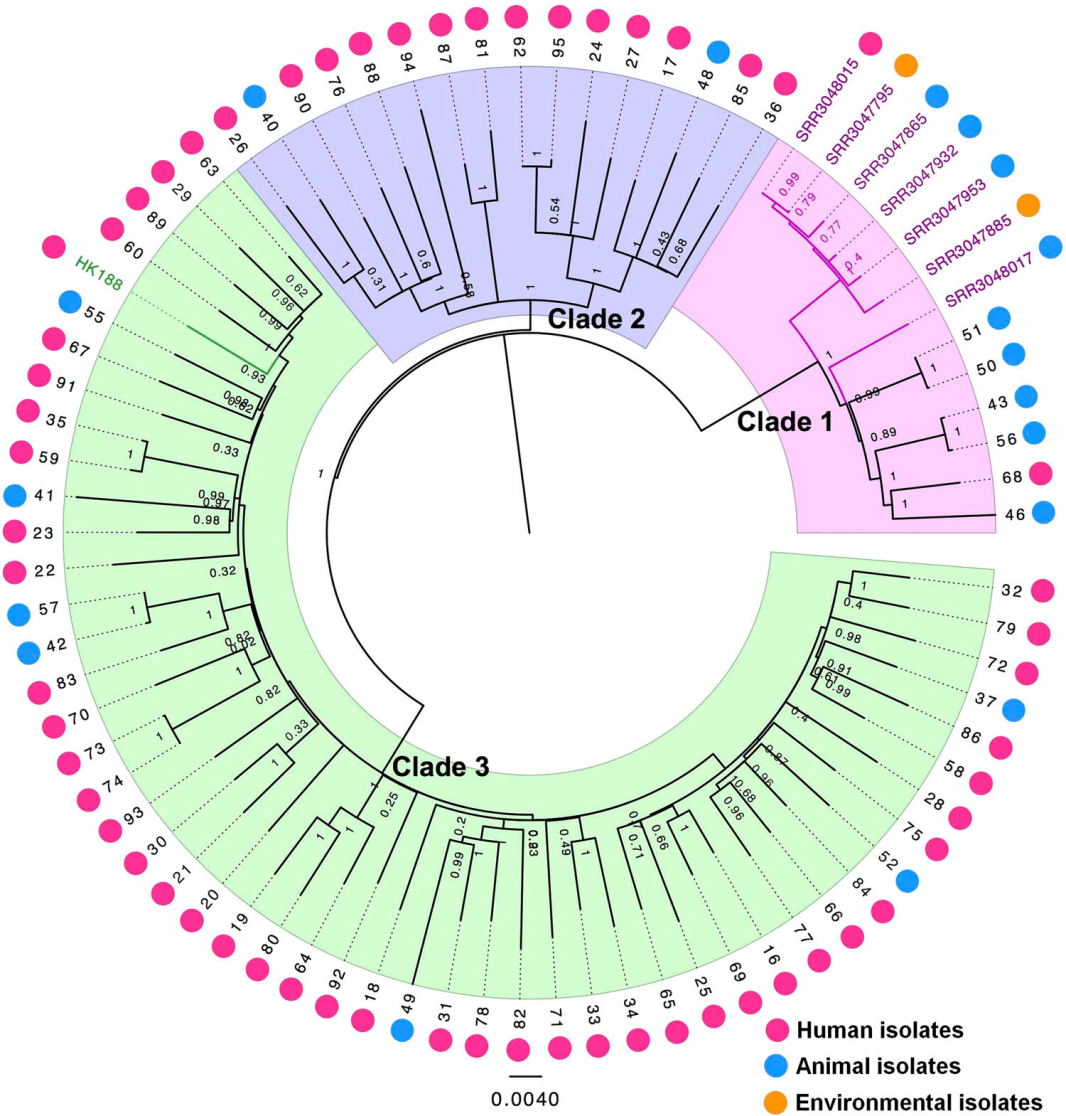
Maximum-likelihood phylogenetic tree of ST188. Seventy-two *S. aureus* ST188 isolates (39 from adult patients, 19 from pediatric patients and 14 from livestock) were used for phylogenetic reconstruction. Relationships are shown with respect to the draft genome of MRSA CUHK_HK188 and seven isolates of MRSA ST188 from North America. Branches are colored by geographic origin of isolates. Black branch: isolates in this study. Pink branch: isolated from North America. Green branch: isolated from Hong Kong. The outer dots indicate the host origin of isolates. Rose-red dots: human-adapted isolates. Blue dots: livestock-adapted isolates. Orange dots: environmental isolates

A total of 72 recoverable ST188 isolates were whole-genome sequenced. The core-genome SNPs were applied for phylogenetic tree reconstruction using maximum likelihood estimation (Fig. 2). All host-adapted ST188 isolates were divided into three major phylogenetic clades, with each supported by 100% bootstrapping. Most of the livestock-associated ST188 were in Clade 1, including four isolates of primate origin LA-MRSA ST188^19^. Human-adapted ST188 were mainly in Clades 2 and 3. Clade 3 accounted for 63.8% (51/80) of ST188 isolates sampled, including human-adapted MRSA CUHK_HK188^20^. Notably, these three clades were all mixtures of samples collected from both humans and livestock. Furthermore, we applied both a parsimony-based method and a Bayesian-based method to estimate the host state of the most common ancestor of ST188 isolates. Both analysis methods indicated a mixed state of the ancestor isolate (Supplementary Figure 2).

**Fig. 3:**
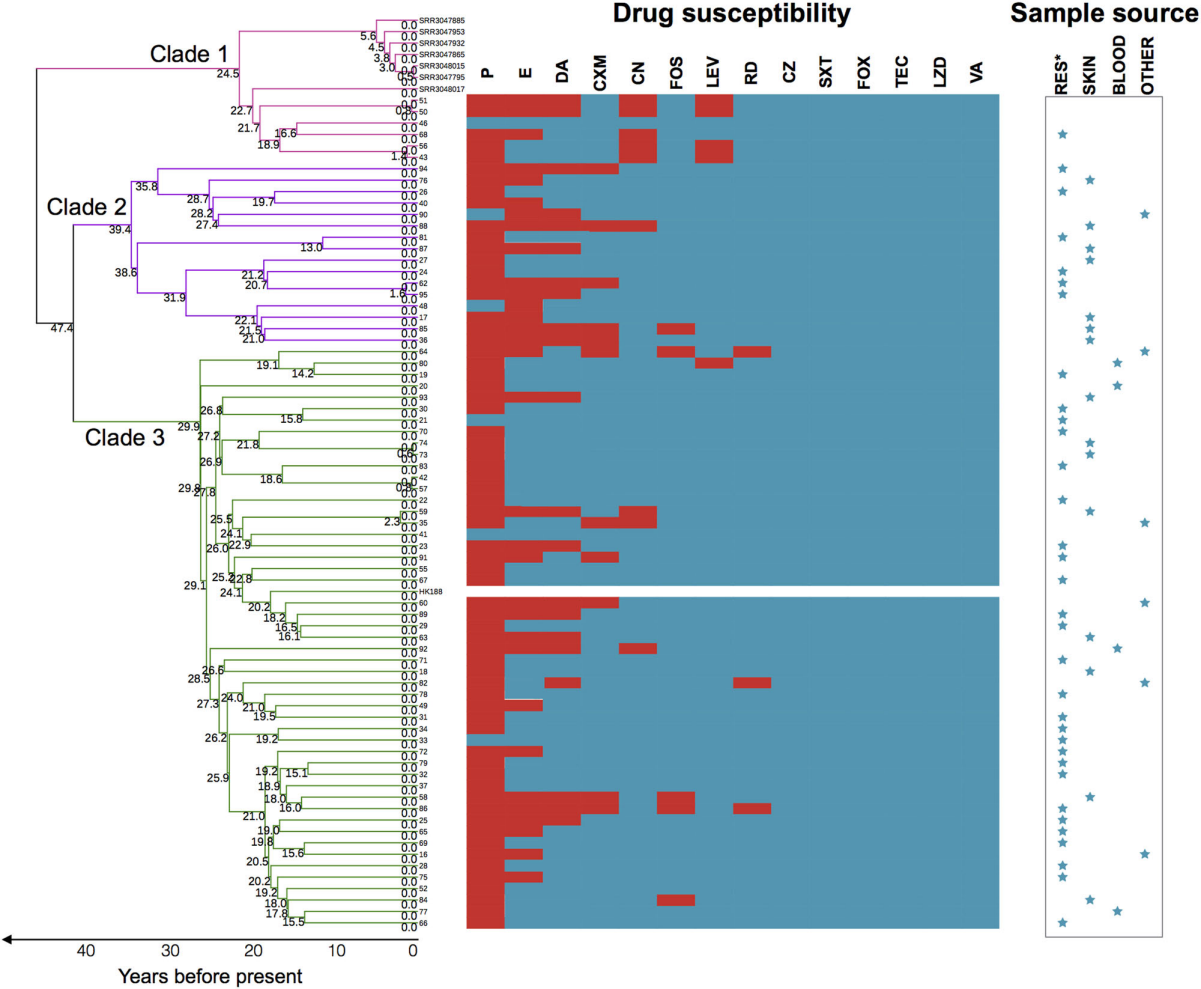
Antibiotic resistance of ST188 isolates from different sources of infection. Branches are colored by different clades and scaled with time (years). See Supplementary Figure 3 for further information about the time scale of the emergence of ST188. The splits represent the resistance (red) or sensitivity (blue) to antibiotics. Sample sources are denoted by blue stars

We further mapped antibiotic susceptibility information, source of infection, and infection type for each isolate to the phylogeny (Fig. 3). All ST188 isolates isolated in Shanghai, China, were MSSA and were susceptible to FOX, SXT, TEC, LZD, OX, and VA. Although the majority of MSSA ST188 in this study were resistant to penicillin (91.7%), they were still susceptible to most of the antibiotics tested. There was no significant antibiotic susceptibility differences between ST188 isolated from livestock and that isolated from humans. ST188 isolates isolated from pediatric and adult patients were well mixed in Clades 2 and 3. In Clade 2, isolates 62 and 95 only differed by 9 SNPs. Similarly, in Clade 3, the pairs 73/74, 42/57 and 59/35 only differed by 2, 4, and 14 SNPs, respectively. Importantly, the isolates in each pair were exclusively sampled from the same hospital, suggesting that these ST188 isolates in each pair were either recently infected from the hospital environment or resulted from patient-to-patient transmission (Fig. 3). Compared with the well-known epidemic host-adapted *S. aureus* ST398, which mainly leads to soft tissue infection^21^, human-adapted MSSA ST188 mainly caused respiratory infection (56.9%).

### Time scale of the recent emergence of *S. aureus* ST188 isolates

To further estimate the time origin of *S. aureus* ST188 isolates, we applied Bayesian-based analysis to the isolates characterized as described above (see Supplementary Methods). Because no longitudinal isolates were obtained and no previous estimate of the mutation rate of ST188 isolates has been reported, we could not precisely calibrate the substitution rate of ST188 isolates. However, under the assumption that substitution rates of subtypes in a given species would mostly be similar, we applied a rough substitution rate that was previously calibrated in *S. aureus* ST398 isolates for Bayesian dating^22^. The results demonstrated that the median time of emergence of all ST188 isolates’ most recent common ancestor (MRCA) was approximately 52.46 years ago (95% HPD, 33.7–82.7), around 1960, pointing to a recent origin of ST188. This time-scaled phylogeny also indicates that ST188 isolates may have first arisen in livestock and were then transmitted to other species. However, the divergence of the three major clades in ST188 occurred more recently: within Clade 1, 24.53 years ago; within Clade 2, 39.41 years ago; and within Clade 3, 29.93 years ago. This pattern indicates that the expansion of ST188 was not simultaneous with its emergence but occurred at least 10–20 years later (Supplementary Figure 3).

### ST188 isolates exhibit high nasal colonization and biofilm formation ability

**Fig. 4:**
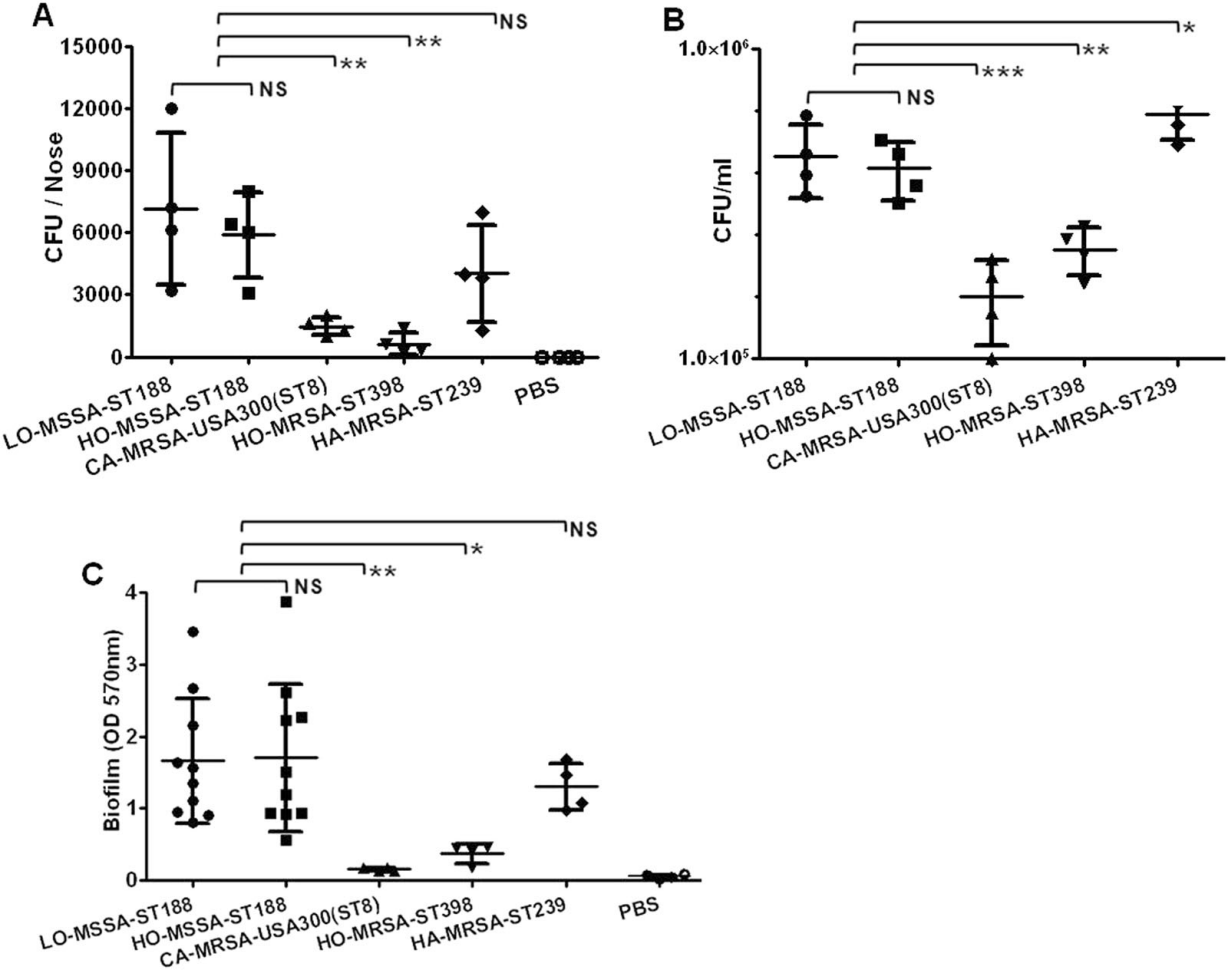
Nasal colonization, cell adhesion, and biofilm formation ability of host-adapted *S. aureus* ST188. **a** The nasal colonization ability of ST188 in mice was compared with that of CA-MRSA USA300 (ST8), HO-MRSA ST398 and HA-MRSA ST239 (4 isolates were randomly selected/lineage). Each mouse (one mouse/isolate) received 1 × 10^8^ colony-forming units (CFUs) in the nares. Control animals (*n* = 4) received only sterile PBS. **b** Adhesion of *S. aureus* to human alveolar epithelial cells A549. Colony counts of adhesive and internalized bacteria on/in A549 epithelial cells after infection. Control group (*n* = 4) received only sterile PBS. **c** Semiquantitative biofilm assays demonstrating the biofilm formation ability of ST188 isolates (10 isolates were randomly selected). *, *P* < 0.05; **, *P* < 0.01; ***, *P* < 0.001; NS, not significant (*P* ≥ 0.05). LO livestock-originated, HO human-originated

The nasal cavity is the predominant location of *S. aureus* colonization in the host body^23^. Previous studies showed that ST188 could be detected from the nares of both human and animals^24,25^. We thus compared the nasal colonization ability in BALB/c mice of ST188 with that of the well-known highly virulent host-adapted *S. aureus* ST398, CA-MRSA USA300 (ST8), and low-virulence HA-MRSA ST239. The results indicated that there was no significant difference either between ST188 isolated from livestock or humans or between ST188 and HA-MRSA ST239. All host-adapted ST188 isolates showed a significantly higher nasal colonization ability than that of CA-MRSA USA300 and host-adapted *S. aureus* ST398 (*P* < 0.01) (Fig. 4a). The epithelial cell adhesion ability of ST188 was much stronger than that of CA-MRSA USA300 (*P* < 0.001) and host-adapted ST398 (*P* < 0.01) (Fig. 4b), further confirming the higher colonization of the ST188 lineage.

*S. aureus* biofilm formation is important for persistent infection, antibiotic resistance and immune evasion^26^. Compared with that of CA-MRSA USA300 and host-adapted ST398, the biofilm formation ability of ST188 was much stronger (*P* < 0.05). There was no significant difference either between ST188 isolated from livestock and humans or between ST188 and HA-MRSA ST239 (Fig. 4c).

**Fig. 5:**
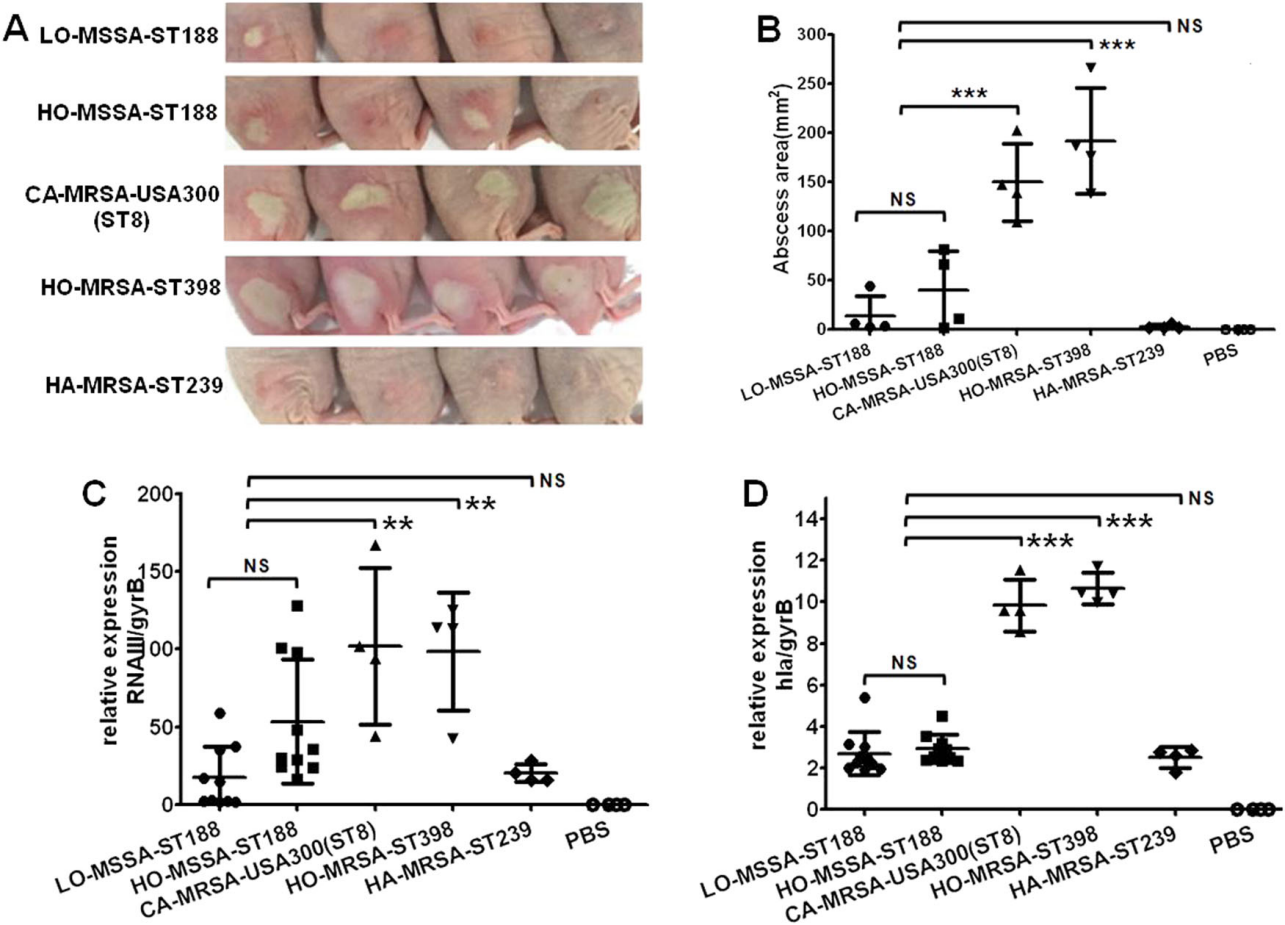
The ability of host-adapted *S. aureus* ST188 to cause invasive infection. **a** Representative abscesses on day 2 after infection (4 isolates were randomly selected/lineage). Each mouse (one mouse/isolate) received 5 × 10^7^ CFUs on the right dorsum by intradermal injection. Control animals (*n* = 4) received only sterile PBS. **b** Abscess areas (=*π* (length × width)/2) on day 2 after infection. **c**, **d** Quantitative RT-PCR analysis of *RNAIII* and *hla* gene expression in randomly selected ST188 (10 isolates were randomly selected), CA-MRSA USA300(ST8), HO-MRSA ST398, and HA-MRSA ST239 (4 isolates were randomly selected/lineage), from cells grown to the late logarithmic growth phase (4 h). **, *P* < 0.01; ***, *P* < 0.001; NS, not significant (*P* ≥ 0.05)

Furthermore, we observed smaller abscesses caused by ST188 isolates compared with those caused by CA-MRSA USA300 (*P* < 0.001) and host-adapted ST398 (*P* < 0.001) using a mouse skin abscess model (Fig. 5a, b); this result is consistent with the lower expression of important virulence factors such as the *Agr* system and α-hemolysin in ST188 isolates (Fig. 5c, d). There was no significant difference between ST188 and HA-MRSA ST239.

### Carrying of virulence genes and phages in the genomes of ST188 isolates

The distribution of virulence genes differed among isolates. There were no significant differences in virulence genes carried between ST188 isolated from livestock and ST188 isolated from humans. Some genes were present in all of the isolates, including *eta*, hemolysin genes, and eight adhesion genes (*cna, ebh, atl, spa, ebp, icaA, icaB*, and *sdrC*) (Supplementary Figure 1B). More than 80% of the ST188 isolates carried the other four adhesion genes (*eap*, *sdrD, sdrE*, and *icaC*), but none of the isolates harbored *tst, sasX, fnbA, fnbB, icaD*, or *pvl*. Most enterotoxin genes (*sea, sed, see, seg, seh, sei, sej, sek, sem, sen, seo, seq ser*, and *seu*) could not be detected in ST188 isolates, but *seb, sec*, and *sel* genes showed lower detection rates in both livestock- and human-adapted ST188 (Gene list see supplementary methods).

A multiplex PCR assay was used to distinguish among the seven most prominent *S. aureus* prophages, Sa1int to Sa7int^27^. Sa3int has been considered to be a molecular marker for distinguishing livestock-adapted from humans-adapted *S. aureus*^28^. In the present study, 100% of human-adapted and 64.3% of livestock-adapted ST188 isolates carried Sa3int. Sa1int was harbored in 21.4% of livestock-adapted ST188 and 32.5% of human-adapted ST188. Sa5int only presented in 14.4% of human-adapted ST188 isolates, and no ST188 isolate harbored Sa2int, Sa4int, Sa6int, or Sa7int (Supplementary Figure 1C).

### Adhesion- and biofilm-formation-related genes are present in multiple copies and are highly expressed in host-adapted ST188 isolates

**Fig. 6:**
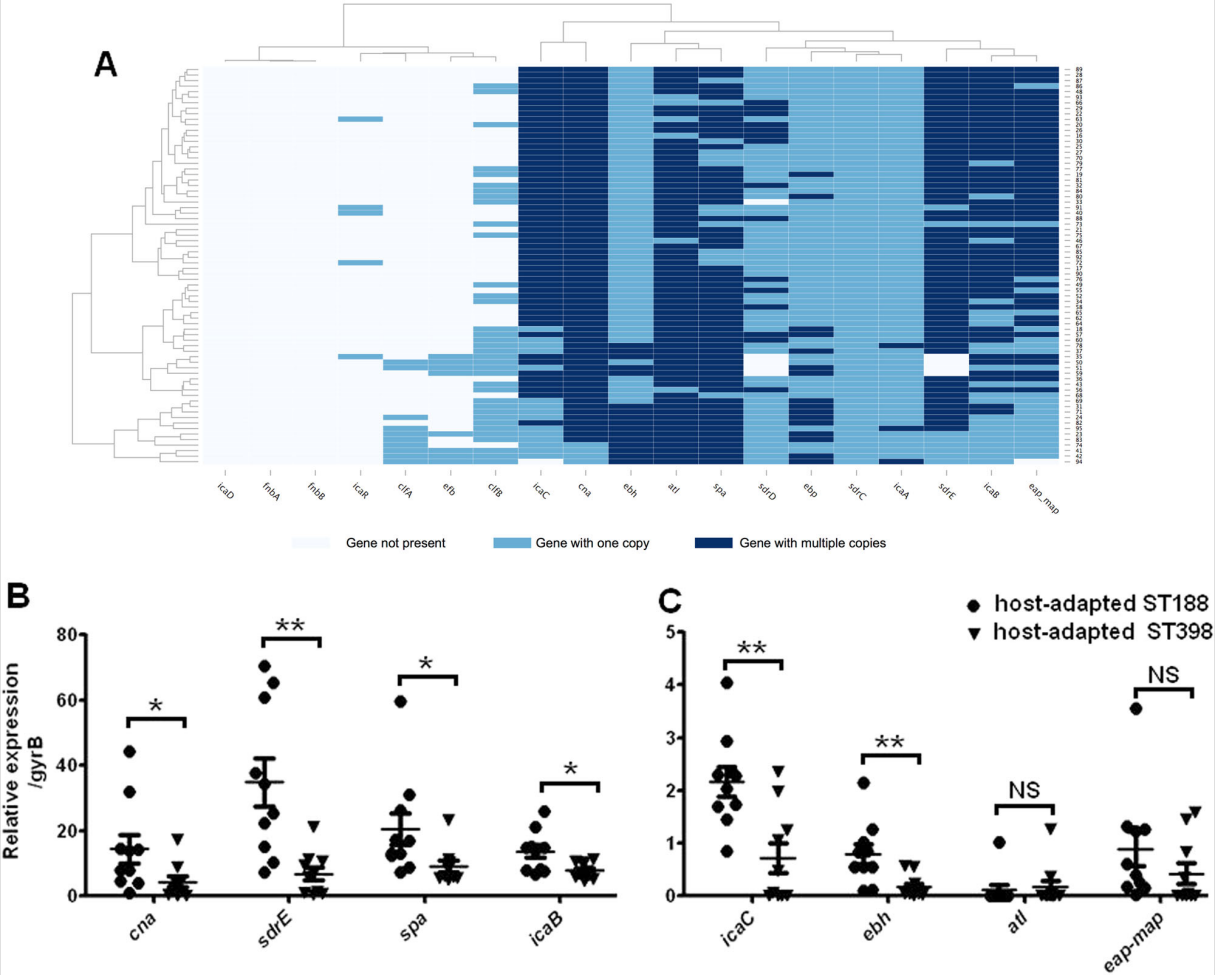
The carrying and expression levels of genes related to adhesion and biofilm formation. **a** The mapping depths of 19 adhesion and biofilm formation genes were detected in all ST188 isolates of this study. A gene with a two-fold higher mapping depth than the average genome sequencing depth was determined as a gene with multiple (≥2) copies (dark blue splits). Genes with one copy were indicated by light blue splits, and genes represented by white splits were determined as absent. **b**, **c** Quantitative RT-PCR analysis of genes with multiple (≥2) copies in host-adapted *S. aureus* ST188 compared with that of host-adapted *S. aureus* ST398 (10 isolates were randomly selected/lineage). *, *P* < 0.05; **, *P* < 0.01; NS, not significant (*P* ≥ 0.05)

Duplicate segments in a genome usually play important roles in bacterial phenotype and virulence. We found many genes related to adhesion and biofilm formation, such as *icaB, icaC, ebh, cna, atl, spa, sdrE*, and *eap* present in multiple copies in some of the ST188 isolates, based on WGS data (Fig. 6a). The expression levels of *icaB, icaC, ebh, spa, cna*, and *sdrE* were higher in ST188 isolates than in host-adapted ST398, as indicated by reverse-transcription polymerase chain reaction (RT-PCR) (Fig. 6b, c). These results are consistent with the robust epithelial cell adhesion and biofilm formation properties of ST188.

## Discussion

Although *S. aureus* ST188 has not yet been described as a global pandemic strain causing hospital-associated infections (HAIs) and community-associated infections (CAIs), it has been increasingly linked to HAIs and CAIs, particularly across the Asia-Pacific region^29^. The strain is more likely to cause HAI in adult patients and CAI in pediatric patients^30,31^. Most reported infections caused by ST188 have been associated with MSSA, but methicillin resistance in ST188 has been reported^19^. Recent epidemiological data show that this strain has a high colonization rate in the nasal cavity of healthy people and animals and could cause infections in various animals^24,25^, implying that *S. aureus* ST188 has the capacity to colonize and cause disease in multiple host species. By characterizing human and bovine *S. aureus* infections between 2012 and 2014, we identified MSSA ST188 as the major lineage causing infections in multiple host species in Shanghai, China. MRSA ST188 was not isolated between 2012 and 2014 in Shanghai, but in our follow-up monitoring, MRSA ST188 causing human infections could be detected, suggesting that ST188 is currently developing antibiotic resistance, thus necessitating continuous monitoring.

ST188 is a double locus variant (DLV) of ST1. However, genetic analysis has indicated large differences between ST188 and ST1, suggesting complex evolutionary processes underlying ST188. WGS is an effective way to analyze the evolution and spread of bacterial strains, but most studies have focused on the globally important host-adapted ST398 instead of ST188. Price *et al*. applied WGS to characterize 89 isolates of ST398 and suggested that livestock-adapted MRSA ST398 originated as MSSA in humans^16^. In this study, 72 isolates of host-adapted MSSA ST188 were whole-genome sequenced. Phylogenetic reconstruction of human and livestock-adapted ST188 suggested that ST188 isolates are readily transmitted among host species. Isolates from livestock did not differ from human-associated isolates and were embedded within them. Additionally, isolates from different hospitals were well-mixed in Clades 2 and 3, and no hospital-specific branch was found. This observation highlighted the potential role of common community commensals in HAI and indicated the recent transmission or continuous infection of ST188 from the same source. Furthermore, our data showed a mixed state of the ancestor isolate, suggesting that ST188 may have infected both humans and livestock before their common ancestor. We further reconstructed the clock-like evolution of ST188 isolates. The median time of clonal emergence was estimated to be ~52.46 years ago, indicating that ST188 isolates apparently first arose in livestock and were then transmitted to humans. However, due to the limited sequencing information of ST188 available worldwide, the evolutionary relationship must be further elucidated.

The predominant Asian CA-SA lineage ST59 can also be isolated from multiple host species^9^. In addition, the worldwide epidemic host-adapted ST398 is also an important strain causing CAI in Asian populations^18^, suggesting that the *S. aureus* lineage causing CAI in humans may be an important risk factor for livestock infection. Most CA-SA clones have gained increased virulence potential via high expression of important virulence components and by causing acute invasive infections, such as severe skin and soft tissue infections (SSTIs)^16^. However, in the present study, the host-adapted ST188 mainly caused HAI in immunocompromised adult inpatients, suggesting that the pathogenesis of ST188 was different from that of ST398. Human-adapted ST398 isolates harbored prophage 3, which contains the immune evasion complex (IEC) genes encoding the chemotaxis inhibitory protein (Chp), staphylococcal complement inhibitor (Scn), and staphylokinase (Sak), is highly human specific and is less prevalent in livestock-adapted ST398^14^. However, in the present study, 64.3% of livestock-adapted ST188 isolates carried prophage 3. We tested 44 virulence genes, but 20 of them (including enterotoxin, *tst, eta, sasX*, and *pvl*) were undetectable in ST188. In addition, no significant difference was found in virulence genes carried between ST188 isolated from livestock and ST188 isolated from humans. The molecular mechanism by which ST188 causes infections in multiple host species requires further clarification.

The *agr* operon encodes a global regulatory system in *S. aureus*, controlling the expression of the genes encoding extracellular virulence factors. Based on the major sequence variations of *agr*, the operon could be divided into four types^32^. Both highly virulent CA-SA USA300 (ST8) and the well-known highly virulent host-adapted *S. aureus* ST398 belong to *agr* type I^33,34^; in this study, all ST188 isolates were also *agr* type I. *Agr* consistently shows high expression levels in highly virulent *S. aureus* strains, but in our study, *RNAIII* (an mRNA with global regulatory properties) showed lower expression levels in ST188 than in USA300 and ST398. Similarly, the abscesses caused by ST188 were much smaller than those caused by USA300 and ST398 in a mice skin abscess model. An in vivo experiment on mice showed that the pathogenicity of ST188 was most likely related to nasal colonization. A robust nasal colonization ability enabled ST188 to successfully colonize and progress to a series of widespread infections. This result was consistent with the strong epithelial cell adhesion and biofilm formation properties of ST188, which was further confirmed by the presence of multiple copies and increased expression of genes related to adhesion and biofilm formation in ST188. The *icaADBC* operon encodes the enzymes necessary for polysaccharide intercellular adhesin (PIA) production, which is the major component of staphylococci biofilms^35^. Ebh is displayed on the surface of *S. aureus* and has been proposed to form a specialized surface structure involved in cellular adhesion. Ebh also plays roles in the determination of *S. aureus* cell size and complement resistance^36^. Cna has been proven to contribute to tissue colonization under various pathological conditions^37^. SPA promotes colonization and immune evasion of *S. aureus*^38^. SdrE is involved in fibrinogen-bridged *S. aureus*–platelet interactions and is required on the bacterial surface for stable adhesion to platelets^39^. Based on the above mentioned evidence, we suspect that ST188 obtained the ability to colonize different hosts by high expression of the factors associated with bacterial adhesion and biofilm formation.

In conclusion, our data provide important insight into the current epidemic status, pathogenicity, transmission, and phylogenetic relationship of host-adapted ST188. Our results reveal that ST188 is an epidemiologically important emerging host-adapted strain. The virulence of ST188 is strongly related to its adhesion and colonization ability, and we have provided evidence of the role of genes related to adhesion and biofilm formation in the colonization of ST188. Further studies are needed to elucidate the molecular mechanisms by which ST188 breaks the barrier of species and is transmitted.

## Materials and methods

### Bacterial isolates and growth conditions

We collected and analyzed 791 clinical isolates from adult patients of Renji Hospital affiliated with Shanghai Jiaotong University and Huashan Hospital affiliated with Fudan University and 158 clinical isolates from pediatric patients of Shanghai Children’s Medical Center affiliated with Shanghai Jiaotong University between 2012 and 2014. Two hundred twelve livestock-associated isolates were isolated from dairy cows with mastitis between 2012 and 2014 in farms near Shanghai, China. *S. aureus* identification was based on Gram staining and classical microbiological tests, and isolates were further characterized using the VITEK 2 Compact GP ID Card (bioMérieux, Marcy l’Etoile, France). *S. aureus* ATCC43300 was used as a quality-control organism. All *S. aureus* isolates were stored at −80 °C.

### Molecular typing of *S. aureus* isolates

MLST of *S. aureus* isolates was performed by detection of seven housekeeping genes (*arcC, aroE, glpF, gmk, pta, tpi*, and *yqiL*)^40^. The sequences of housekeeping genes were submitted to the *S. aureus* MLST database (http://www.mlst.net). The sequence of the polymorphic X region of the *spa* gene was submitted to the *S. aureus spa* type database (http://www.spaserver.ridom.de)^41^. The *agr* type was detected by multiplex PCR as previously described^32^.

### DNA extraction and WGS

Some isolates were contaminated and then discarded, or they died during long-term preservation or during transportation. Ultimately, 72 *S. aureus* ST188 isolates (39 from adult patients, 19 from pediatric patients and 14 from livestock) were used for WGS. WGS was carried out using the HiSeq 4000 sequencing platform (Illumina Inc., San Diego, CA) with a 2 × 150 bp read length. (Majorbio Bio-Pharm Technology, Shanghai, China). The fragment size for the pair-end libraries was 500 bp. The raw data were filtered before assembly, and clean reads were obtained after removing the adapter sequences and low-quality sequences (*Q* < 20). In addition, sequences containing more than 10% ambiguous N bases or sequences shorter than 30 bp in length were also removed. The Illumina sequences generated and used in this study available in the Sequence Read Archive (SRA) (http://www.ncbi.nlm.nih.gov/sra) under study accession numbers SRR6227128 to SRR6227199.

### SNP calling and phylogenetic analysis

The Sickle tool was used for WGS data trimming. The whole-genome sequence of the *S. aureus* MW2 strain (ST1, GenBank accession code: BA000033.2) was used as the reference template for read mapping. The genome of MRSA ST188 CUHK_HK188 (GenBank accession no.: JFFV00000000), isolated from a human in Hong Kong^20^, and seven isolates of MRSA ST188 (GenBank accession no.: SRR3048015 from the human nasal cavity; SRR3047795 and SRR3047865 from the environment; and SRR3047932, SRR3047953, RR3047885 and SRR3048017 from primates) isolated from North America were added for comparison^19^ (detailed information is provided in Supplementary Methods).

### Detecting the presence of virulence-associated genes

We detected the presence of 44 virulence-associated genes. The DNA sequences of virulence genes were used as a genomic template for mapping the sequencing reads of each *S. aureus* ST188 isolate individually (details in Supplementary Methods). Furthermore, we checked the mapping depth of the 19 adhesion and biofilm formation genes. A gene with a two-fold higher mapping depth than the average genome sequencing depth was determined as a gene with multiple (≥2) copies.

### Phage integrase multiplex PCRs

Prophages carried by ST188 isolates were detected by using a previously described method^27^. A multiplex PCR scheme was used to detect the seven most important *S. aureus* integrase families, Sa1int to Sa7int. Primers were designed according to the phage integrase genes, and multiplex PCR was performed using a multiplex PCR kit (Qiagen, Germany).

### Mouse skin abscess and nasal colonization models

All animal experiments were performed following the guidelines for the Care and Use of Laboratory Animals of the Chinese Association for Laboratory Animal Sciences. The animal protocol was approved by the Committee on the Ethics of Animal Experiments of Renji Hospital, School of Medicine, Shanghai Jiaotong University, Shanghai, China. Outbred, immune-deficient hairless female mice (4–6 weeks old) were used for the abscess model. BALB/c female mice (4–6 weeks old) were used for the nasal colonization model (details in Supplementary Methods).

### Adhesion of *S. aureus* to human alveolar epithelial cells A549

Human alveolar epithelial cells A549 were cultured in DMEM medium with fetal bovine serum (FBS, 10%) at 37 °C and 5% CO_2_. Bacteria were grown to the mid-logarithmic growth phase and washed twice with DMEM medium. A549 cells and bacteria were used at a 1:10 ratio (MOI = 10) and incubated for 2 h. Culture supernatants were discarded, and cells were washed three times with sterile PBS to remove non-adherent bacteria. A549 cells were lysed by the addition of 0.1% deoxysodium cholate solution. Bacterial CFU were enumerated by serial dilutions of epithelial cell lysates and plating onto trypticase soy agar (TSA) plates.

### Quantitative reverse-transcription (RT) PCR

The expression of adhesion and biofilm formation genes in ST188 was detected by RT-PCR. We chose ST398 as the reference group because ST398 is an important host-species-adaptable *S. aureus* lineage with high virulence but low biofilm formation ability^18,21^. Complementary DNA was synthesized from total RNA using the QuantiTect reverse transcription system (Qiagen) according to the manufacturer’s instructions. Complementary DNA samples were amplified using the QuantiTect SYBR green PCR kit (Qiagen). Reactions were performed using a 7500 Sequence Detector (Applied Biosystems). We used purified chromosomal DNA at concentrations of 0.005–50 ng/ml to form a standard curve. All quantitative reverse-transcription polymerase chain reaction (qRT-PCR) experiments were performed in duplicate with *gyrB* as a control.

### Statistical analysis

Unpaired two-tailed Student’s *t*-tests were performed to analyze statistical significance. All data were analyzed using GraphPad Prism, version 6.0, and *P* values <0.05 were deemed statistically significant.

## Electronic supplementary material

Supplementary Data

Figure S1

Figure S2

Figure S3
