# Supplementary material for: Phylogenetic analysis and virulence determinant of the host-adapted Staphylococcus aureus lineage ST188 in China
Source: Emerg Microbes Infect. 2018 Mar 29;7:45. doi: 10.1038/s41426-018-0048-7 (PMC5874244; doi:10.1038/s41426-018-0048-7)
Supplement: Supplementary file 1 — Supplementary Data [file 41426_2018_48_MOESM1_ESM.doc]

**Supplementary Data**

**Phylogenetic analysis and virulence determinant of the host-adapted *Staphylococcus aureus* lineage ST188 in China**

Yanan Wang, Qingyun Liu, Qian Liu, Qianqian Gao, Huiying Lu, Hongwei Meng, Yihui Xie, Qian Huang, Xiaowei Ma, Hua Wang, Juanxiu Qin, Qiong Li, Tianming Li, Min Li

**Supplementary Methods**

**Antibiotic susceptibility tests**

Antibiotic susceptibility was tested by the standard disk diffusion method in accordance with the Clinical and Laboratory Standards Institute guidelines (CLSI, 2014). The antibiogram classifications were made on susceptibility to 14 antimicrobials: gentamycin (CN), penicillin (P), cefazolin (CZ), erythromycin (E), clindamycin (DA), sulfamethoxazole-trimethoprim (SXT), fosfomycin (FOS), rifampicin (RD), levofloxacin (LEV), cefuroxime (CXM), teicoplanin (TEC), linezolid (LZD), vancomycin (VA) and cefoxitin (FOX).

**SNP calling and phylogenetic analysis**

Maximum-likelihood method was used to reconstruct the phylogenetic trees using MEGA of version 6.0 under the general time-reversible (GTR) nucleotide substitution model and 500 bootstrap replicates were performed. Sequencing reads were mapped to reference genome using Bowtie2 of version 2.2.9. SAMtools was used for SNP calling with mapping quality greater than 30. Fixed mutations (frequency ≥ 75%) were identified using VarScan of version 2.3.9 with at least 5 reads supporting and no strand bias. The core genome sequence was created based on the consensus sequences of all *S. aureus* isolates, and was defined as the nucleotide sites that were shared by all strains^1^. After removing nucleotide sites that showing gaps or with extremely low sequencing depth/quality, a total of 2,090,037 genomic sites were defined as the core genome sequence and SNPs detected in this core sequence were used for further analysis.

**Bayesian based coalescent analysis**

The WGS of 72 *S. aureus* ST188 isolates were used for phylogenetic reconstruction and a total of 20,760 concatenated genome-wide SNPs were used for phylogenetic analyses. We estimated the dates of most recent common ancestors of all ST188 isolates and each major using BEAST (v1.8.0)^2^. We used GTR model for these datasets. The XML-input file was modified to specify the number of invariant sites in the MTBC genomes. For *S. aureus* genome substitution rate, we applied a previously characterized substitution rate of 1.49×10^-6^ substitutions per site per year (95% highest posterior density (HPD) interval 9.44×10^-7^ to 1.97×10^-6^) in *S. aureus* ST398 (ref. 1). We used an uncorrelated lognormal distribution for the substitution rate, and we used a constant population size for the tree priors. We run 3 chains of 5×10^7^ generations and sampled every 10,000 to assure independent convergence of the chains, the first 10% of which were discarded as a burn-in. Convergence was assessed using Tracer (v1.6.0)[^3^](#_ENREF_3) and Phylogenetic trees were visualized using Figtree (v1.4.2) (<http://tree.bio.ed.ac.uk/software/figtree/>). The software RASP[^4^](#_ENREF_4) that implemented both Bayesian and parsimony (S-DIVA) approaches was used for estimating the ancestral ranges all ST188 strains sampled here. For the parsimony-based analyses(S-DIV), a maximum of two ancestral areas per node were allowed for range reconstruction. For the Bayesian-based analyses (BBM), five different chains during 500 thousand generations were run.

**Mouse skin abscess model**

Mice were anesthetized with 2,2,2-Tribromoethanol (dissolved in 2-Methyl-2-butanol) and inoculated with 100 μl PBS containing 5×10^7^ live *S. aureus* (mid-logarithmic phase) or PBS alone in the right flank by subcutaneous injection. Abscess length (L) and width (W) values were measured to calculate the area (A) of abscesses at 24-h after inoculation. A=π (L× W)/2[^5^](#_ENREF_5).

**Mouse nasal colonization model**

All mice were received ampicillin (100μg/ml) in their drinking water. 1 ×10^8^ CFUs (mid-logarithmic phase) in 10 µl of PBS or PBS alone, was pipetted slowly into the nares of the anesthetized mice with 2,2,2-Tribromoethanol. Mice were killed and evaluated for nasal carriage of *S. aureus* on the third day after inoculation. The nasal region was cleaned by 70% ethanol, and nasal tissues were homogenized. The total number of *S. aureus* was assessed by plating 100 µl diluted nasal suspensions on TSB agar containing ampicillin (100 µg/ml)[^6^](#_ENREF_6).

**Detecting the presence of virulence associated genes**

The DNA sequences of virulence genes were used as genomic template for mapping of sequencing reads of each *S. aureus* ST188 isolates individually. If the sequencing reads were successfully mapped to a gene of interest with the average mapping depth above 50% of that of the whole genome and the gene coverage was above 80%, this gene was determined as presence and otherwise determined as absence. Sequences of virulence genes were obtained from the NCBI nucleotide database, including 17 staphylococcal enterotoxin genes (*sea, seb, sec, sed, see, seg, seh, sei, sej, sek, sel, sem, sen, seo, seq, ser, seu*), toxic shock syndrome toxin (*tst*), exfoliative toxin gene (*eta*), 4 hemolysin genes (*hla, hlb, hld, hlg*), surface protein (*sasX*), Panton-Valentine leukocidin (*pvl*), and 19 adhesion and biofilm formation genes (*clfA, clfB, fnbA, fnbB, spa, atl, ebh, cna, ebp, eap-map, efb, icaA, icaB, icaC, icaD, icaR, sdrC, sdrD* and *sdrE*).

**Semiquantitative biofilm assay**

The supernatant of *S. aureus* culture was discarded after incubated. The organisms covered the bottom of 96-well tissue culture plates, and be fixed by Bouin’s fiative for 1 h. Then, wells were washed gently with sterile PBS. Organisms in the wells were then stained with crystal violet, and the floating stain was washed off with slow- running water. After drying, the stained biofilm was read with an ELISA autoreader (BioRad) at 570 nm.

**Supplementary References**

1 Uhlemann, A. C. *et al.* Evolutionary Dynamics of Pandemic Methicillin-Sensitive Staphylococcus aureus ST398 and Its International Spread via Routes of Human Migration. *mBio* **8**, doi:10.1128/mBio.01375-16 (2017).

2 Drummond, A. J. & Rambaut, A. BEAST: Bayesian evolutionary analysis by sampling trees. *BMC evolutionary biology* **7**, 214, doi:10.1186/1471-2148-7-214 (2007).

3 Rambaut, A. & Drummond, A. Tracer: a program for analysing results from Bayesian MCMC programs such as BEAST & MrBayes (University of Edinburgh, UK, 2003).

4 Yu, Y., Harris, A. J., Blair, C. & He, X. RASP (Reconstruct Ancestral State in Phylogenies): a tool for historical biogeography. *Molecular phylogenetics and evolution* **87**, 46-49, doi:10.1016/j.ympev.2015.03.008 (2015).

5 Wang, R. *et al.* Identification of novel cytolytic peptides as key virulence determinants for community-associated MRSA. *Nature medicine* **13**, 1510-1514, doi:10.1038/nm1656 (2007).

6 Li, M. *et al.* MRSA epidemic linked to a quickly spreading colonization and virulence determinant. *Nature medicine* **18**, 816-819, doi:10.1038/nm.2692 (2012).

**Supplementary Figure 1**. *Spa* types, virulence genes and phages prevalence in ST188 isolated from different host-species. *A*, *Spa* type was determined based on the polymorphic X region of the *spa* gene, and the *spa* types of all ST188 isolates were detected to compare the distributions in different host species. *B*, Virulence-associated genes (44 genes), including 17 staphylococcal enterotoxin genes, toxic shock syndrome toxin (*tst*), exfoliative toxin gene (*eta*), 4 hemolysin genes, surface protein (*sasX*), Panton-Valentine leukocidin (*pvl*), and 19 adhesion and biofilm formation genes, were detected and their prevalence determined. Genes are not shown in this figure if they do not exist in the genome of ST188. *C,* A multiplex PCR was used to distinguish among the seven most prominent *S. aureus* prophages, Sa1int to Sa7int, in ST188 from different host species.

**Supplementary Figure 2**. The host state of the most common ancestor of ST188 isolates. The software RASP, which implements both Bayesian and parsimony-based (S-DIVA) approaches, was used to estimate the ancestral ranges of all ST188 isolates sampled.

**Supplementary Figure 3.** Time scale of the emergence of host-adapted *S. aureus* ST188. For the *S. aureus* genome substitution rate, we applied a previously characterized substitution rate of 1.49×10^-6^ substitutions per site per year (95% highest posterior density (HPD) interval 9.44×10^-7^ to 1.97×10^-6^). The CUHK_HK188 and seven isolates of MRSA ST188 from North America were included. Branches are colored by different clades and scaled with time (years).
