# Supplementary figures and images for: Phylogenetic analysis and virulence determinant of the host-adapted Staphylococcus aureus lineage ST188 in China
Source: Emerg Microbes Infect. 2018 Mar 29;7:45. doi: 10.1038/s41426-018-0048-7 (PMC5874244; doi:10.1038/s41426-018-0048-7)

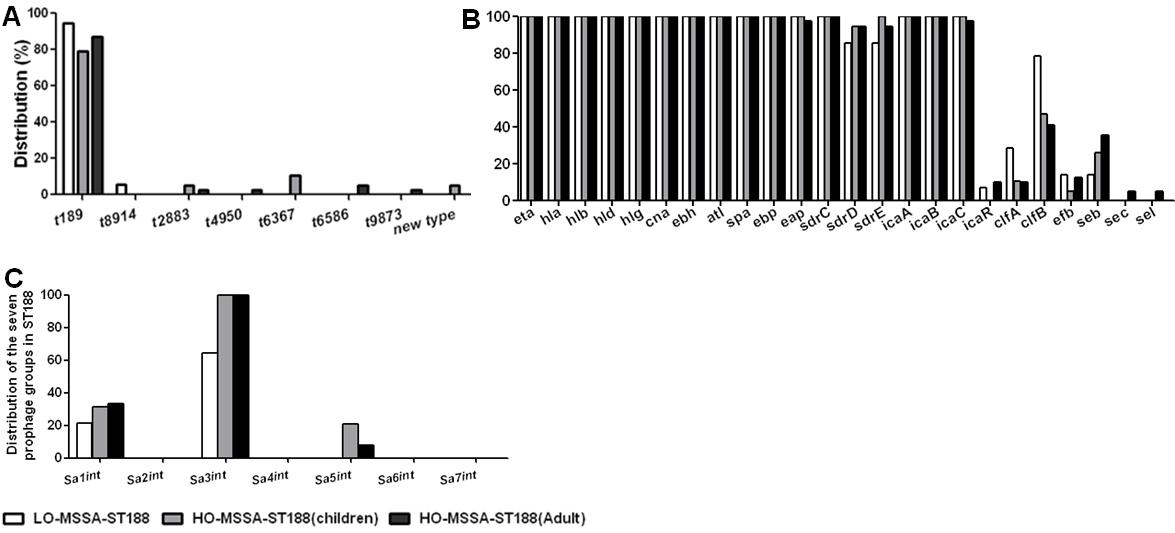

Supplement: Supplementary file 2 — Figure S1 [file 41426_2018_48_MOESM2_ESM.jpg]

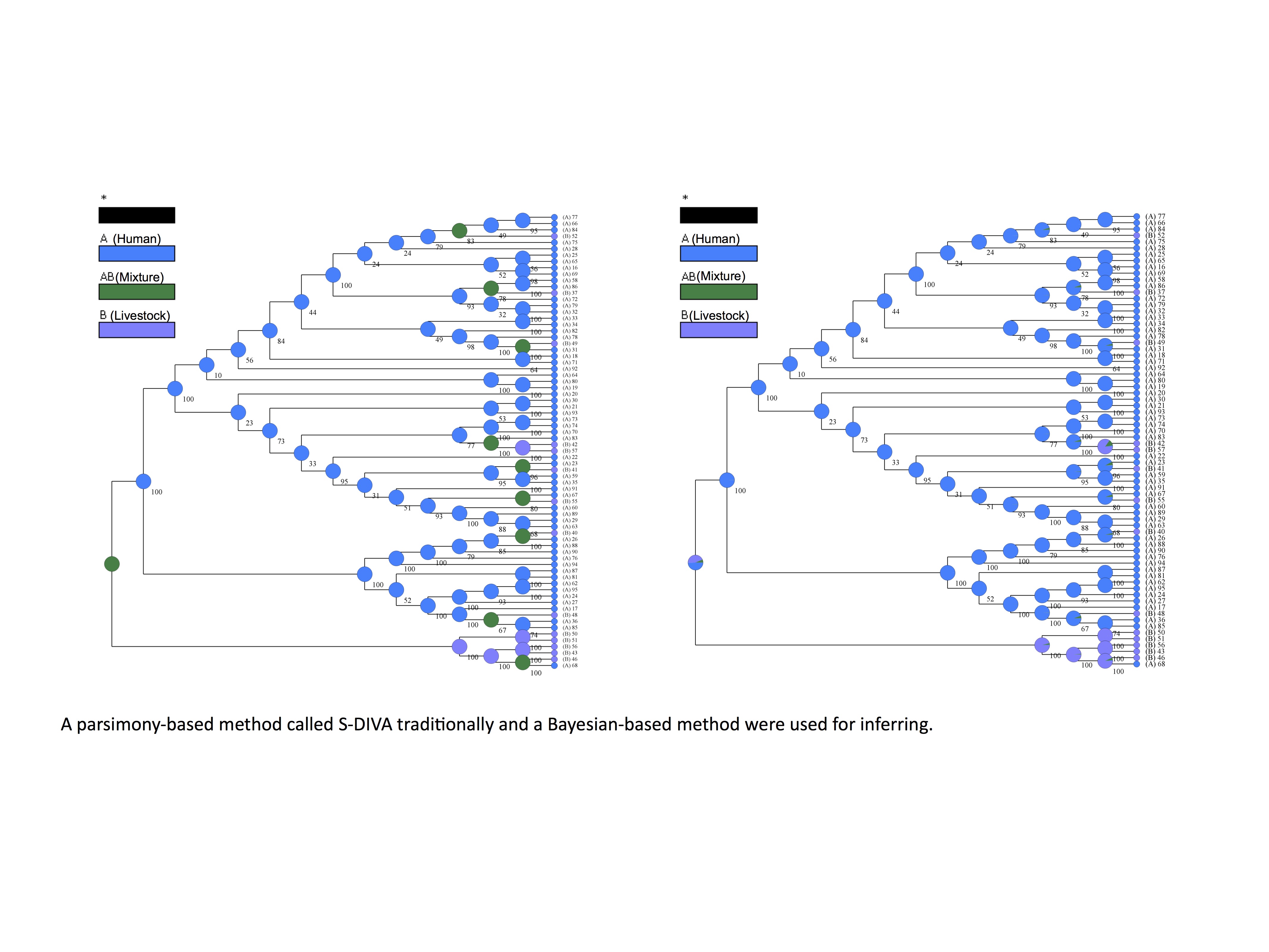

Supplement: Supplementary file 3 — Figure S2 [file 41426_2018_48_MOESM3_ESM.jpg]

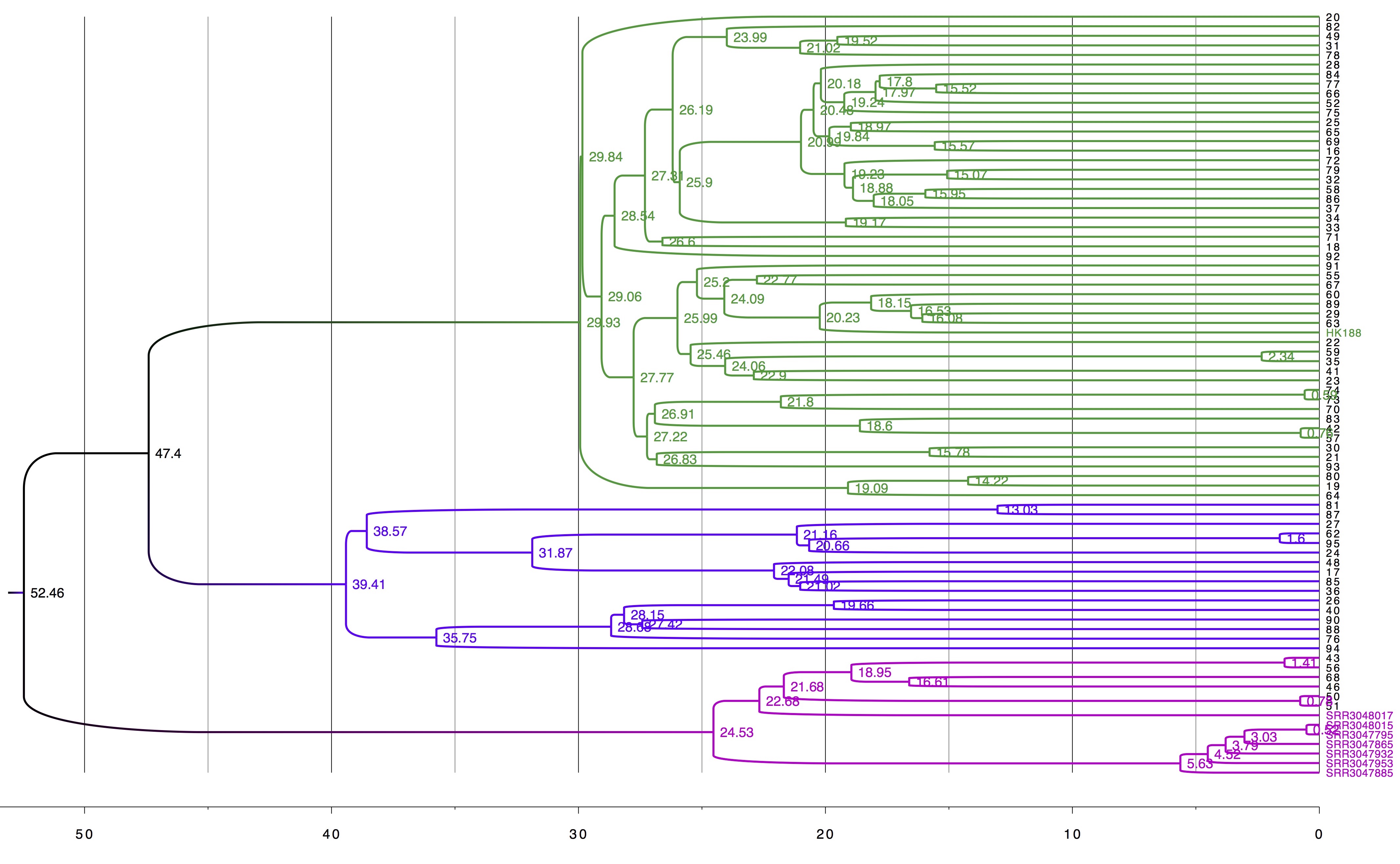

Supplement: Supplementary file 4 — Figure S3 [file 41426_2018_48_MOESM4_ESM.jpg]
